# Hernia width explains differences in outcomes between primary and incisional hernias: a prospective cohort study of 9159 patients

**DOI:** 10.1007/s10029-020-02340-1

**Published:** 2020-11-23

**Authors:** L. Verstoep, G. H. J. de Smet, D. Sneiders, L. F. Kroese, G.-J. Kleinrensink, J. F. Lange, J.-F. Gillion, The Hernia-Club Members

**Affiliations:** 1 Department of Surgery, Erasmus University Medical Center, Rotterdam, The Netherlands; 2 Department of Surgery, Reinier de Graaf Gasthuis, Delft, The Netherlands; 3 Department of Neuroscience, Erasmus University Medical Center, Rotterdam, The Netherlands; 4 Department of Surgery, IJsselland Ziekenhuis, Capelle aan den IJssel, The Netherlands; 5 Unité de Chirurgie Viscérale et Digestive, Hôpital Prive d’Antony, Antony, France; 6 Department of Surgery, Erasmus University Medical Center, PO BOX 2040, Room Ee-173, Dr. Molewaterplein, 3000 CA Rotterdam, The Netherlands

**Keywords:** Incisional hernia, Primary ventral hernia, Hernia surgery, Postoperative outcomes

## Abstract

**Purpose:**

Data on primary (PH) and incisional hernias (IH) are often pooled, even though several studies have illustrated that these are different entities with worse outcomes for IHs. The aim of this study is to validate previous research comparing PHs and IHs and to examine whether hernia width is an important contributor to the differences between these hernia types.

**Methods:**

A registry-based, prospective cohort study was performed, utilizing the French Hernia Club database. All patients undergoing PH or IH repair between September 8th 2011 and May 22nd 2019 were included. Baseline, hernia and surgical characteristics, and postoperative outcomes were collected. Outcomes were analyzed per width category (≤ 2 cm, 3–4 cm, 5–10 cm and > 10 cm).

**Results:**

A total of 9159 patients were included, of whom 4965 (54%) had PH and 4194 (46%) had IH. PHs and IHs differed significantly in 12/15 baseline characteristics, 9/10 hernia and surgical characteristics, and all outcomes. Overall, complications and re-interventions were more common in patients with IH. After correcting for width, the differences between PH and IH were no longer significant, except for medical complications, which were more common after IH repair compared to PH.

**Conclusion:**

After correcting for hernia width, most outcomes do not significantly differ between PH and IH, indicating that not hernia type, but hernia width is an important factor contributing to the differences between PH and IH.

**Electronic supplementary material:**

The online version of this article (10.1007/s10029-020-02340-1) contains supplementary material, which is available to authorized users.

## Introduction

In 2009 the European Hernia Society (EHS) formulated separate classifications for primary (PH) and incisional abdominal wall hernias (IH), as these were considered to be two different entities [1]. However, in hernia research, data on PH and IH is still often pooled [2–5]. In the last decade, several articles have been published comparing PH and IH, which concluded that these hernias are significantly different entities in terms of baseline characteristics, surgical and hernia characteristics and outcomes [6–9]. Köckerling et al*.* [6] and Stirler et al*.* [8] concluded that patients with IH had significantly worse outcomes with higher postoperative complication rates and higher recurrence rates. Köckerling et al*.* [6] subsequently concluded that PH and IH were treated differently in terms of mesh location and open or laparoscopic procedure. Stirler et al*. *[8] also found a higher total conversion rate and longer hospital stay for patients with IH compared to patients with PH. In 2018 Kroese et al*.* [7] performed a prospective cohort study of 4565 patients, based on the French Hernia Club database. Besides having similar conclusions as previous research, they found that PH and IH differed significantly in terms of baseline characteristics.

Whether the different outcomes are caused by underlying differences in etiology is not yet completely understood. Other possible causes are the higher incidence of comorbidities in IH patients or differences in hernia width [7]. Larger IHs are more prone to unfavorable outcomes than smaller IHs [10]. On average, IHs are significantly larger than PHs, and the difference in postoperative outcomes between PH and IH may be due to the difference in hernia width.

Since the study of Kroese et al. [7] the number of patients included in the French Hernia Club registry has doubled. Therefore, the aims of this study are to validate previous results of Kroese et al*.* by repeating the study with double power and to determine whether hernia width is an important contributing factor causing the differences in patient characteristics and postoperative outcomes between PH and IH.

## Methods

This prospective cohort study was performed within the French Hernia Club registry. The Hernia Club registry is approved by the French ‘Commission Nationale de l’Informatique et des Libertés’ (CNIL; registration number 1993959v0) and complies to the General Data Protection Regulation. Additional consent by participants and institutional review board approval were not required since this is a registry-based study and patient data is anonymized, which is in accordance with the Dutch and French national ethical standards. This study was conducted according to the European Registry of Abdominal Wall Hernias (EuraHS) recommendations and follows the STROCSS criteria and STROBE (Strengthening the Reporting of Observational Studies in Epidemiology) guidelines for observational studies [11–13].

### Study design

This is a registry-based, prospective cohort study within the Hernia Club database. All adults undergoing ventral hernia repair of both PH and IH between the 8th of September 2011 until the 22nd of May 2019 in the French Hernia Club registry were compared. Parastomal hernias were excluded. To compare PH and IH and to validate previous research on the subject, baseline, hernia and surgical characteristics, and postoperative outcomes were collected. To determine whether hernia width explains these differences, baseline characteristics and postoperative outcomes were compared for different width categories. The constructed width categories are as follows: 0 up to and including 2 cm, 3 up to and including 4 cm, 5 up to and including 10 cm, and wider than 10 cm. Hernia width was only available in whole centimeters. These categories were defined roughly based on the combined width categories in the EHS classification for PH and IH, as both hernia types have a different hernia width classification [1].

### Hernia Club registry

The Hernia Club registry is a collaborative, prospective, anonymized online database of all surgical procedures for primary and incisional hernias. All surgical procedures were performed by French surgeons specialized in abdominal wall surgery. The Charter of Quality must be signed and accepted by each participating surgeon, which states that: ‘‘all input must be registered in a consecutive, un-selected, and exhaustive manner and in real time’’. 191 parameters, including the pre-, peri- and post-operative periods, are prospectively collected by the operating surgeon and blinded, independent clinical research associates using online forms. In case of any discrepancies the medical records are checked. Patients consent to random peer review of medical charts to ensure high-quality data. The parameters collected in this registry are in accordance with the EuraHS international online platform and the European Hernia Society (EHS) classification of primary and incisional abdominal wall hernias [1, 14].

### Data collection

Patient baseline characteristics extracted from the registry include age, sex, body mass index (BMI), smoking habits, diabetes mellitus, corticosteroid use, history of radiotherapy, chemotherapy, abdominal aortic aneurysm, connective tissue disorder, history of abdominal wall hernia, hiatal hernia, family history of hernia and American Society of Anesthesiologists (ASA) classification. Hernia characteristics extracted from the database comprised the type of hernia, hernia width, hernia length and preoperative symptoms. Surgical characteristics extracted from the database were planned or emergency surgery, duration of operation, open or laparoscopic approach, primary suture or mesh closure, mesh location, Altemeier wound classification and antibiotic treatment [15]. Extracted postoperative data included the duration of admission, intra-operative complications (including penetrating digestive tract wound, bladder wound, severe bleeding, and general complications like an embolism or infarction), wound complications (including SSIs, SSOs and SSOPIs), surgical complications (including occlusion of the small intestine, peritonitis, intraperitoneal abscesses, retro- or subperitoneal hematoma, abdominal wall hematoma, mesh migration, abdominal wall compartment syndrome and early recurrence), medical complications (including veinitis/lymfangitis, thrombophlebitis, deep venous thrombosis, urinary retention, pneumonia, cardiac rhythmic disorders, acute coronary syndrome and neurological complications) and re-intervention [16].

### Statistical analyses

SPSS 26.0 (IBM Corp. Released 2019. IBM SPSS Statistics for Windows, version 26.0. IBM Corp, Armonk, NY, USA) was used for all statistical analyses. Continuous variables were tested for normal distribution with the Shapiro–Wilk test and *Q*–*Q* plots. Non-normally distributed continuous variables are presented as medians with interquartile ranges. Categorical variables are presented as absolute numbers with percentages. Mann–Whitney *U* (continuous data) and chi-squared tests (categorical data) were used to compare PH and IH patients. To demonstrate the overall comparison and to avoid emphasis on one particular factor, it was chosen to do this by performing univariable analysis without additional multivariable analysis. Baseline patient characteristics and outcomes were also compared per width category with Mann–Whitney *U* and chi-squared tests. Variables with less than fifteen patients in either of the groups were excluded from the analysis per width category. *P* values < 0.05 were considered statistically significant.

## Results

A total 9159 patients were included. Of these patients, 4965 (54%) underwent surgery for PH repair and 4194 (46%) patients underwent IH repair.

### Baseline patient characteristics

All baseline patient characteristics are presented in Online Resource 1. Patients with PH and IH differed significantly for 12 of 15 baseline characteristics studied.

### Hernia and surgical characteristics

Hernia and surgical characteristics are presented in Online Resource 2. Patients with PH and IH were statistically significantly different for nine out of ten hernia and surgical characteristics, except for emergency surgery. Hernia width and length were significantly larger in patients with IH (median width 2.0, interquartile range 1.0) compared to patients with PH (median width 5.0, interquartile range 4.0, *p* < 0.001).

### Postoperative outcomes

Postoperative outcomes are presented in Online Resource 3. All complications and the incidence of re-interventions were more common in patients with IH compared to patients with PH.

### Hernia width

The number of patients in each width category are presented in Table 1. Primary hernias were more likely to be small. Most PHs were 0–2 cm wide (*n* = 3270, 82.4%), compared to 442 (10.9%) IHs (*p* < 0.001). IHs were significantly larger than PHs; 1501 (36.9%) IHs were 2–4 cm wide versus 556 (14.3%) PHs (p < 0.001), 1683 (41.4%) IHs were 5–10 cm wide versus 116 (2.9%) PHs (*p* < 0.001). Sixteen (0.4%) patients with PH had a hernia wider than 10 cm, compared to 438 (10.8%) patients with IH (*p* < 0.001).

**Table 1:** Width per hernia type

	Primary hernia (*n* = 4965)	Incisional hernia (*n* = 4194)	*p* value
0–2 cm	3270 (82.4)	442 (10.9)	** < 0.001**
3–4 cm	566 (14.3)	1501 (36.9)	** < 0.001**
5–10 cm	116 (2.9)	1683 (41.4)	** < 0.001**
> 10 cm	16 (0.4)	438 (10.8)	** < 0.001**
Missing	997 (20.1)	130 (3.1)	

Statistically significant *p* values are in bold (*p*<0.05)

Data are *n* (%)

### Baseline characteristics per width category

Baseline characteristics per width category are presented in Online Resource 4. Overall, patients with IH were significantly older compared to patients with PH (median age of 67.0 years versus 57.0 years, *p* < 0.001). The median age of patients with IH sized 3–4 cm and 5–10 cm was significantly higher than for patients with PH (66.0 years versus 63.0 years, *p* < 0.001; 67.0 years versus 62.5 years, *p* < 0.001, respectively). Significantly more patients with PH sized 0–2 and 3–4 cm were male, compared to patients with IH (63.1% versus 40.0%, *p* < 0.001; 63.3% versus 48.6%, *p* < 0.001, respectively). There were no differences in the proportions of male sex for larger hernias. Overall the incidence of diabetes mellitus was significantly higher for patients with IH, 7.1% for PH versus 13.8% for IH (*p* < 0.001). However, there was no significant difference in the incidence of diabetes per width category. History of chemo- or immunotherapy was more common in patients with IH (1.3%; 0.9%; 2.6% for 0–2, 3–4 and 5–10 cm, respectively) versus patients with PH (3.4%; 3.9%; 8.1% for 0–2 (*p* = 0.001), 3–4 (*p* = 0.001) and 5–10 cm (*p* = 0.034), respectively), with an overall incidence of 6.7% for patients with an IH and 1.2% for patients with PH (*p* < 0.001). Anti-coagulant use was more common in patients with IH with a width of 2–4 and 5–10 cm (IH 18.5% versus PH 12.1%, *p* = 0.001; IH 17.9%, PH 9.6%, *p* = 0.022, respectively). There was no difference in anticoagulant use in small hernias (0–2 cm).

### Outcomes per width category

Outcomes per width category are presented in Table 2. The admission duration was significantly higher for patients with IH, regardless of the width category. The incidence of intra-operative complications was not significantly different for any of the width categories. Overall, intra-operative complications were more common among patients with IH (IH 2.2%; PH 0.5%, *p* < 0.001). The incidence of any wound or surgical complication per width category was similar for IH and PH. With increasing hernia width the complication rates increased. Overall, the incidence of wound complications (PH 2.9%; IH 7.8%, *p* < 0.001) and surgical complications (PH 0.7%; IH 4.4%, *p* < 0.001) was higher for patients undergoing IH repair compared to patients undergoing PH repair. Medical complications were more common among patients with an IH sized 2–4 and 5–10 cm (PH 1.6%; IH 4.5%, *p* = 0.003; PH 1.0%; IH 6.2%, *p* = 0.035, respectively). Per width category, there was no significant difference in the incidence of re-intervention for PH and IH. Overall, the incidence of re-intervention is higher for patients with IH (IH 2.6%; PH 0.8%, *p* < 0.001).

**Table 2:** Outcomes per width category

	Primary hernia	*n* missing (%)	Incisional hernia	*n* missing (%)	*p* value
Admission duration, days					
0–2 cm	0.0 (1.0)	347 (10.6)	0.0 (2.0)	43 (9.7)	** < 0.001**
3–4 cm	0.0 (2.0)	70 (12.4)	2.0 (4.0)	186 (12.4)	** < 0.001**
5–10 cm	2.0 (4.0)	24 (20.7)	5.0 (4.0)	243 (14.4)	** < 0.001**
> 10 cm	0.0 (6.0)	1 (6.3)	7.0 (5.0)	80 (18.3)	** < 0.001**
Total	0.0 (1.0)	522 (10.5)	3.0 (5.0)	578 (13.8)	** < 0.001**
Intra-operative complications					
0–2 cm	16 (0.5)	194 (5.9)	3 (0.7)	18 (4.1)	0.623
3–4 cm	4 (0.7)	32 (5.7)	16 (1.1)	83 (5.5)	0.458
5–10 cm	2 (1.7)	7 (6.0)	39 (2.3)	74 (4.4)	0.697
> 10 cm	0 (0.0)	0 (0.0)	26 (6.1)	11 (2.5)	0.309
Total	25 (0.5)	278 (5.6)	87 (2.2)	210 (5.0)	** < 0.001**
Wound complications within 30 days					
0–2 cm	87 (3.0)	358 (10.9)	12 (3.1)	49 (11.1)	0.943
3–4 cm	25 (5.0)	63 (11.1)	73 (5.4)	144 (9.6)	0.726
5–10 cm	8 (8.4)	21 (18.1)	142 (9.2)	139 (8.3)	0.799
> 10 cm	1 (6.3)	0 (0.0)	62 (15.3)	32 (7.3)	0.321
Total	131 (2.9)	524 (10.6)	297 (7.8)	394 (9.4)	** < 0.001**
Surgical complications within 30 days					
0–2 cm	18 (0.6)	377 (11.5)	4 (1.0)	50 (11.3)	0.364
3–4 cm	6 (1.2)	67 (11.8)	33 (2.4)	151 (10.1)	0.099
5–10 cm	4 (4.2)	21 (18.1)	77 (5.0)	149 (8.9)	0.725
> 10 cm	1 (6.3)	0 (0)	39 (9.7)	37 (8.4)	0.634
Total	32 (0.7)	552 (11.1)	167 (4.4)	415 (9.9)	** < 0.001**
Medical complications within 30 days					
0–2 cm	52 (1.8)	345 (10.6)	11 (2.8)	51 (11.5)	0.159
3–4 cm	8 (1.6)	62 (11.0)	61 (4.5)	141 (9.4)	**0.003**
5–10 cm	1 (1.0)	18 (15.5)	96 (6.2)	134 (8.0)	**0.035**
> 10 cm	0 (0.0)	0 (0.0)	53 (13.1)	32 (7.3)	0.122
Total	68 (1.5)	504 (10.2)	230 (6.0)	387 (9.2)	** < 0.001**
Re-intervention^a^					
0–2 cm	21 (0.7)	407 (12.4)	3 (0.8)	57 (12.9)	0.922
3–4 cm	9 (1.8)	74 (13.1)	19 (1.4)	168 (11.2)	0.533
5–10 cm	1 (1.1)	23 (19.8)	40 (2.6)	166 (9.9)	0.353
> 10 cm	1 (6.3)	0 (0.0)	32 (8.0)	38 (8.7)	0.800
Total	35 (0.8)	5964 (12.0)	99 (2.6)	457 (10.9)	** < 0.001**

Statistically significant *p* values are in bold (*p*<0.05)

Data are mean (interquartile range) for continuous variables and *n* (%) for categorical variables

^a^Re-intervention includes both surgical and radiological re-intervention

## Discussion

To this day, data on PH and IH is often pooled, even though several studies suggest that these hernias are different entities [6–9]. The present analysis of 9159 patients of the prospective French Hernia Club database validates previous research stating that PH and IH are significantly different in terms of most baseline, surgical and hernia characteristics, and postoperative outcomes. After correcting for hernia width, however, most outcomes no longer showed significant differences, indicating that not the type of hernia, but the hernia width is an important factor that largely contributes to these differences.

IH repair is often considered as a procedure with an increased risk of intra-operative and postoperative complications compared to PH repair. Based on the data in the present study this assumption seems only partly correct. The emphasis should be on hernia size instead of hernia type. In accordance with previous studies by Heniford et al*.* [17] and Helgstrand et al. [18], the present study shows that with increasing hernia size, the complication rate increases, as well as the re-intervention rate. Both intra-operative-, wound- and surgical complication rates, as well as the re-intervention rate, are not statistically significantly different when comparing PH and IH per width category. The width distribution is skewed, with a larger average width for IH compared to PH, indicating that not the type of hernia, but the hernia width is an important factor causing the differences between IH and PH found in this study and the previous study by Kroese et al*.* [7]. Repair of small IHs should be viewed as low-risk procedures, as the complication rates are similar to the rates of small PHs. In relation to this, large PHs should not be underestimated, as these have as many intra-operative, wound- and surgical complications as large IHs. However, this is not the case for medical complications, which are more common in patients with IH with a width of 3–4 and 5–10 cm. Patients with IH in these width categories are significantly older and have a higher incidence of a history of chemo- or immunotherapy and a higher incidence of anti-coagulant use compared to patients with PH. This indicates a higher incidence of cancer patients and/or patients with vascular disease and possibly a worse overall condition, making them more susceptible to medical complications. As a possible result of this, the admission duration was significantly longer for patients with IH compared to patients with PH, regardless of any width category.

Although worse outcomes of IH mostly appear to be the result of larger width, hernia type and hernia width cannot be viewed separately from each other. PH and IH have different etiologies. PH seems to be a congenital condition or the result of a prolonged period of increased intra-abdominal pressure, IH being the result of failed wound healing of often large incisions, possibly explaining why IH is in general larger compared to PH. There is extensive evidence that in IH changes in the homeostasis of the extracellular matrix (ECM), especially switches in collagen phenotypes, are prominent [19]. It is likely that there are similar changes in the ECM of PH. However, to our knowledge, there is no study examining ECM changes in which data on IH and PH is not pooled. Future research examining whether differences between the ECM of PH and IH explains differences in hernia size and outcomes is necessary.

A meta-analysis by Stabilini et al*.* [9] found that IH has a higher recurrence rate than PH. However, the question remains whether this is still the case after correcting for hernia width. Hernia recurrence is not reported in the present study due to insufficient data on PH recurrence in the Hernia Club database. A probable explanation for this is the fact that PH has a smaller average width, less frequently having concomitant defects at the surgery and a shorter operative time, resulting in a simpler procedure and, subsequently, a shorter post-operative follow-up period compared to patients with IH, resulting in less available data on PH recurrence [9]. As both the formation of IH and hernia recurrence are the result of failed wound healing, it seems logical that hernia recurrence is more common in IH. Nevertheless, several studies have shown that larger hernias have a higher recurrence rate compared to smaller hernias, forming another possible explanation of the higher recurrence rates of IH of around 30% [18, 20–22].

### Limitations

This study is limited by the fact that the data are not randomized, which may cause a potential risk of confounding by indication. However, since all patients who underwent ventral hernia repair were included in the registry, no selection has been made in patient inclusion and this study is, therefore, a good representation of the general patient population. Still, in the present study, 46% of the patients had IH which is much higher than the expected 30% found in previous research, and could potentially indicate a risk of selection bias [23]. A possible explanation for this higher percentage is the fact that de Hernia Club registry is the collaborative work of surgeons with a special interest in hernia surgery, and might, therefore, attract more complicated patients. The expertise of the surgeons might also explain the relatively low rate of complications.

The incidence of PH with a width of > 10 cm is low (*n* = 16). Therefore, statistically reliable conclusions on hernias wider > 10 cm cannot be made, since this study is underpowered for this width category. However, the other width categories showed an increase in complication and re-intervention rates with increasing width. It is, therefore, likely that this can be extrapolated for hernias with a width of > 10 cm.

Hernia width is missing in 20.1% of PH, whereas this is only 3.1% in IH. As a result, only 80% of data on PH was available for the analysis per width category. This might be because ultrasounds or CT scans are less often performed for the clinical workup before PH repair. This may be more commonly performed for IH, since this hernia type is often larger, more complex, and sometimes has multiple fascial defects.

## Conclusion

The distribution of hernia width is skewed; IH is generally larger than PH. Wider hernias have worse outcomes, which is an important factor explaining the differences in outcomes between PH and IH. In practice, small IH repair can be viewed as low risk as repair of small PH and repair of wide PH should be regarded as complex as repair of wide IH. Reporting the width in hernia research is strongly recommended.

## Electronic supplementary material

Below is the link to the electronic supplementary material.

Supplementary file1 (PDF 422 KB)

## Data Availability

All data is derived from the Hernia-Club registry and raw data would remain confidential and would not be shared. More information about the Hernia-Club registry is found in the materials and method section and at https://www.club-hernie.com/.

## Code availability

SPSS version 26.0 has been used to do all calculations in this article.
