# Supplementary material for: Hernia width explains differences in outcomes between primary and incisional hernias: a prospective cohort study of 9159 patients
Source: Hernia. 2020 Nov 23;25(2):463–9. doi: 10.1007/s10029-020-02340-1 (PMC8055619; doi:10.1007/s10029-020-02340-1)
Supplement: Supplementary file 1 — Supplementary file1 (PDF 422 KB) [file 10029_2020_2340_MOESM1_ESM.pdf]

Supplementary material

Online Resource 1. Baseline patient characteristics

	Primary hernia (n = 4965)	N missing (%)	Incisional hernia (n =4194)	N missing (%)	p-value
**Age in years**	57 (22)	0	67 (19)	0	**<0.001**
**Male sex**	3017 (60.8)	0	1970 (47.0)	0	**<0.001**
**BMI, kg/m2**	27.1 (7)	47 (0.9)	28.4 (7)	48 (1.1)	**<0.001**
**Smoking**	1086 (22.3)	98 (2.0)	730 (18.2)	178 (4.2)	**<0.001**
**Diabetes mellitus**	349 (7.1)	64 (1.3)	567 (13.8)	75 (1.8)	**<0.001**
**Corticosteroid use**	100 (2.0)	64 (1.3)	108 (2.6)	75 (1.8)	0.067
**Pelvic radiotherapy**	19 (0.4)	64 (1.3)	89 (2.2)	75 (1.8)	**<0.001**
**Chemo- or immunotherapy**	59 (1.2)	64 (1.3)	274 (6.7)	75 (1.8)	**<0.001**
**AAA**	2 (0.04)	33 (0.7)	22 (0.5)	24 (0.6)	**<0.001**
**Connective tissue disorder**	5 (0.1)	33 (0.7)	5 (0.1)	24 (0.6)	0.790
**Anticoagulant use or coagulopathy**	421 (8.6)	64 (1.3)	712 (17.3)	75 (1.8)	**<0.001**
**Ascites**	32 (0.7)	67 (1.3)	19 (0.5)	54 (1.3)	0.219
**History of abdominal wall hernia**	709 (14.4)	33 (0.7)	1708 (41.0)	24 (0.6)	**<0.001**
Inguinal hernia	535 (10.8)	33 (0.7)	513 (12.3)	24 (0.6)	**0.030**
Primary ventral hernia	153 (3.1)	33 (0.7)	705 (16.9)	24 (0.6)	**<0.001**
Incisional hernia	34 (0.7)	33 (0.7)	696 (16.7)	24 (0.6)	**<0.001**
**Family history of hernia**	203 (4.1)	33 (0.7)	40 (1.0)	24 (0.6)	**<0.001**
**ASA class ≥ 3**	676 (13.7)	46 (0.9)	1195 (28.7)	29 (0.7)	**<0.001**

Data are mean (interquartile range) for continuous variables and n (%) for categorical variables.
BMI: body mass index; AAA: aneurysm of the abdominal aorta; ASA: American Society of Anesthesiologists.

Online Resource 2. Hernia and surgical characteristics

Hernia characteristics	Primary hernia (n = 4965)	N missing (%)	Incisional hernia (n = 4194)	N missing (%)	p-value
**Hernia width, cm**	2.0 (1.0)	997 (20.1)	5.0 (4.0)	130 (3.1)	**<0.001**
**Hernia length, cm**	2.0 (1.0)	1002 (20.2)	5.0 (6.0)	184 (4.4)	**<0.001**
**Symptoms**		37 (0.7)		34 (0.8)	**<0.001**
Asymptomatic	1051 (21.3)		610 (14.7)		
Symptomatic[a]	3877 (78.6)		3550 (85.3)		
**Surgical characteristics**					
**Emergency surgery**	180 (3.7)	113 (2.3)	170 (4.1)	79 (1.9)	0.305
**Duration of operation, min**	20.0 (15.0)	340 (6.8)	48.0 (60.0)	269 (6.4)	**<0.001**
**Laparoscopic surgery**	1629 (33.8)	147 (3.0)	1191 (29.2)	113 (2.7)	**<0.001**
**Primary suture**	1374 (28.9)	203 (4.1)	363 (8.9)	108 (2.6)	**<0.001**
**Mesh placement**	3388 (71.1)	203 (4.1)	3723 (91.1)	108 (2.6)	**<0.001**
Intraperitoneal	2440 (72.0)		2408 (64.7)		
Sublay	907 (26.8)		1208 (32.4)		
Onlay	36 (1.1)		96 (2.6)		
**Altemeier wound classification**		116 (2.3)		76 (1.8)	**<0.001**
Clean	4656 (96.0)		3833 (93.1)		
Clean contaminated	163 (3.4)		198 (4.8)		
Contaminated	24 (0.5)		60 (1.5)		
Dirty	6 (0.1)		27 (0.7)		
**Antibiotic treatment**		113 (2.3)		90 (2.1)	**<0.001**
None	2233 (46.0)		699 (17.0)		
Prophylactic	2567 (52.9)		3197 (77.9)		
Therapeutic	52 (1.1)		208 (5.1)		

Data are mean (interquartile range) for continuous variables and n (%) for categorical variables.
[a] *Symptomatic includes discomfort, pain and/or incarceration.*

Online Resource 3. Postoperative outcomes

	Primary hernia (n = 4965)	N missing (%)	Incisional hernia (n = 4194)	N missing (%)	p-value
**Admission duration, days**	0.0 (1.0)	552 (10.5)	3.0 (5.0)	578 (13.8)	**<0.001**
**Intra-operative complications**	25 (0.5)	278 (5.6)	87 (2.2)	210 (5.0)	**<0.001**
**Wound complication within 30 days**	131 (2.9)	524 (10.6)	297 (7.8)	394 (9.4)	**<0.001**
**Surgical complication within 30 days**	32 (0.7)	552 (11.1)	163 (4.3)	415 (9.9)	**<0.001**
**Medical complication within 30 days**	68 (1.5)	504 (10.2)	230 (6.0)	387 (9.2)	**<0.001**
**Re-intervention**[a]	35 (0.8)	594 (12.0)	99 (2.6)	457 (10.9)	**<0.001**

Data are mean (interquartile range) for continuous variables and n (%) for categorical variables.
[a] Re-intervention includes both surgical and radiological re-interventions.

Online Resource 4. Baseline characteristics per width category

	Primary hernia	N missing (%)	Incisional hernia	N missing (%)	p-value
**Age (years)**					
0-2 cm	58.0 (21)	0 (0.0)	59.0 (25)	0 (0.0)	0.425
3-4 cm	63.0 (21)	0 (0.0)	66.0 (20)	0 (0.0)	**<0.001**
5-10 cm	62.5 (24)	0 (0.0)	67.0 (17)	0 (0.0)	**<0.001**
>10 cm	66.0 (24)	0 (0.0)	69.0 (18)	0 (0.0)	0.140
Total	57.0 (22)	0 (0.0)	67.0 (19)	0 (0.0)	**<0.001**
**Sex (male)**					
0-2 cm	2063 (63.1)	0 (0.0)	177 (40.0)	0 (0.0)	**<0.001**
3-4 cm	358 (63.3)	0 (0.0)	730 (48.6)	0 (0.0)	**<0.001**
5-10 cm	50 (43.1)	0 (0.0)	775 (46.0)	0 (0.0)	0.538
>10 cm	10 (62.5)	0 (0.0)	229 (52.3)	0 (0.0)	0.421
Total	3017 (60.8)	0 (0.0)	1970 (47.0)	0 (0.0)	**<0.001**
**BMI, kg/m^2**					
0-2 cm	27.36 (7)	28 (0.9)	25.84 (7)	4 (0.9)	**<0.001**
3-4 cm	30.43 (8)	12 (2.1)	28.30 (7)	10 (0.7)	**<0.001**
5-10 cm	29.38 (8)	1 (0.9)	29.12 (8)	23 (1.4)	0.615
>10 cm	27.93 (5)	0 (0.0)	29.38 (8)	8 (1.8)	0.690
Total	27.12 (7)	47 (0.9)	28.41 (7)	48 (1.1)	**<0.001**
**Smoking**					
0-2 cm	699 (21.8)	66 (2.0)	117 (27.2)	12 (2.7)	**0.012**
3-4 cm	94 (17.0)	14 (2.5)	253 (17.5)	53 (3.5)	0.815
5-10 cm	18 (15.9)	3 (2.6)	253 (15.8)	79 (4.7)	0.965
>10 cm	2 (12.5)	0 (0)	85 (20.2)	17 (3.9)	0.450
Total	1086 (22.3)	98 (2.0)	730 (18.2)	178 (4.2)	**<0.001**
**Diabetes mellitus**					
0-2 cm	222 (6.9)	43 (1.3)	27 (6.2)	6 (1.4)	0.593
3-4 cm	65 (11.8)	14 (2.5)	187 (12.6)	22 (1.5)	0.597
5-10 cm	16 (13.9)	1 (0.9)	263 (16.0)	37 (2.2)	0.558
>10 cm	1 (6.3)	0 (0.0)	71 (16.5)	8 (1.8)	0.273
Total	349 (7.1)	64 (1.3)	567 (13.8)	75 (1.8)	**<0.001**
**Use of corticosteroids**					
0-2 cm	57 (1.8)	43 (1.3)	12 (2.8)	6 (1.4)	0.155
3-4 cm	13 (2.4)	14 (2.5)	34 (2.3)	22 (1.5)	0.940
5-10 cm	0 (0.0)	1 (0.9)	48 (2.9)	37 (2.2)	0.063
>10 cm	0 (0.0)	0 (0.0)	12 (2.8)	8 (1.8)	0.498
Total	100 (2.0)	64 (1.3)	108 (2.6)	75 (1.8)	0.067
**History of pelvic radiotherapy**					
0-2 cm	11 (0.3)	43 (1.3)	7 (1.6)	6 (1.4)	**<0.001**
3-4 cm	3 (0.5)	14 (2.5)	24 (1.6)	22 (1.5)	0.059
5-10 cm	0 (0.0)	1 (0.9)	37 (2.2)	37 (2.2)	0.104
>10 cm	0 (0.0)	0 (0.0)	8 (1.9)	8 (1.8)	0.582
Total	19 (0.4)	64 (1.3)	89 (2.2)	75 (1.8)	**<0.001**
**History of chemo- or immunotherapy**					
0-2 cm	42 (1.3)	43 (1.3)	15 (3.4)	6 (1.4)	**0.001**
3-4 cm	5 (0.9)	14 (2.5)	57 (3.9)	22 (1.5)	**0.001**

5-10 cm	3 (2.6)	1 (0.9)	133 (8.1)	37 (2.2)	**0.034**
>10 cm	0 (0.0)	0 (0.0)	53 (12.3)	8 (1.8)	0.135
Total	59 (1.2)	64 (1.3)	274 (6.7)	75 (1.8)	**<0.001**
**Use of anticoagulants**					
0-2 cm	290 (9.0)	43 (1.3)	42 (9.6)	6 (1.4)	0.659
3-4 cm	67 (12.1)	14 (2.5)	274 (18.5)	22 (1.5)	**0.001**
5-10 cm	11 (9.6)	1 (0.9)	295 (17.9)	37 (2.2)	**0.022**
>10 cm	3 (18.8)	0 (0.0)	84 (19.5)	8 (1.8)	0.938
Total	421 (8.6)	64 (1.3)	712 (17.3)	75 (1.8)	**<0.001**
**History of ascites**					
0-2 cm	14 (0.4)	45 (1.4)	0 (0.0)	1 (0.2)	0.166
3-4 cm	10 (1.8)	10(1.8)	7 (0.5)	16 (1.1)	**0.003**
5-10 cm	3 (2.6)	2 (1.7)	9 (0.5)	24 (1.4)	**0.008**
>10 cm	0 (0,0)	0 (0,0)	3 (0,7)	8 (1.8)	0.737
Total	32 (0.7)	67 (1.3)	19 (0.5)	54 (1.3)	0.219
**History of abdominal wall hernia**					
Inguinal hernia					
0-2 cm	346 (10.7)	25 (0.8)	92 (20.9)	1 (0.2)	**<0.001**
2-4 cm	60 (10.7)	5 (0.9)	201 (13.5)	9 (0.6)	0.092
5-10 cm	12 (10.4)	1 (0.9)	157 (9.4)	10 (0.6)	0.710
>10 cm	2 (12.5)	0 (0)	53 (12.1)	1 (0.2)	0.964
Total	535 (10.8)	33 (0.7)	513 (12.3)	24 (0.6)	**0.030**
Primary ventral hernia					
0-2 cm	89 (2.7)	25 (0.8)	88 (20.0)	1 (0.2)	**<0.001**
2-4 cm	31 (5.5)	5 (0.9)	286 (19.2)	9 (0.6)	**<0.001**
5-10 cm	6 (5.2)	1 (0.9)	247 (14.8)	10 (0.6)	**0.004**
>10 cm	0 (0.0)	0 (0)	68 (15.6)	1 (0.2)	0.087
Total	153 (3.1)	33 (0.7)	705 (16.9)	24 (0.6)	**<0.001**
Incisional hernia					
0-2 cm	24 (0.7)	25 (0.8)	27 (6.1)	1 (0.2)	**<0.001**
2-4 cm	5 (0.9)	5 (0.9)	202 (13.5)	9 (0.6)	**<0.001**
5-10 cm	1 (0.9)	1 (0.9)	254 (21.2)	10 (0.6)	**<0.001**
>10 cm	0 (0.0)	0 (0)	89 (20.4)	1 (0.2)	**0.044**
Total	34 (0.7)	33 (0.7)	696 (16.7)	24 (0.6)	**<0.001**
**Family history of hernia**					
0-2 cm	109 (3.4)	25 (0.8)	7 91.6)	1 (0.2)	**0.046**
2-4 cm	13 (2.3)	5 (0.9)	20 (1.3)	9 (0.6)	0.117
5-10 cm	1 (0.9)	1 (0.9)	10 (0.6)	10 (0.6)	0.718
>10 cm	2 (12.5)	0 (0)	2 (0.5)	1 (0.2)	**<0.001**
Total	203 (4.1)	33 (0.7)	40 (1.0)	24 (1.6)	**<0.001**
**ASA class ≥ 3**					
0-2 cm	419 (12.9)	32 (1.0)	85 (19.3)	2 (0.5)	**<0.001**
3-4 cm	126 (22.5)	5 (0.9)	386 (25.9)	9 (0.6)	0.111
5-10 cm	31 (27.0)	1 (0.9)	527 (31.6)	13 (0.8)	0.303
>10 cm	4 (25.0)	0 (0.0)	172 (39.4)	1 (0.2)	0.247
Total	676 (13.7)	46 (0.9)	1195 (28.7)	29 (0.7)	**<0.001**

Data are mean (interquartile range) for continuous variables and n (%) for categorical variables.
BMI: body mass index; ASA: American Society of Anesthesiologists.
